# Using Short-Term Concentration Measures and Intelligence in Rehabilitation Settings

**DOI:** 10.1100/tsw.2006.233

**Published:** 2006-10-23

**Authors:** Bruce D. Kirkcaldy

**Affiliations:** International Centre for the Study of Occupational and Mental Health, Düsseldorf, Germany

**Keywords:** short-term concentration, intelligence, psychopathology, letter cancellation task, rehabilitation, Germany

## Abstract

Psychological assessment of cognitive functioning among clinical groups that include psychiatric and geriatric patients is a difficult task. This study examines the interrelationship between intelligence and concentrative ability for a group of psychiatric patients. Intellectual and concentrative ability were assessed among a group of 85, predominantly schizophrenic, patients (mean age 32 years) from a Sheltered Workshop (GWN) and neurological-psychiatric institutionalized care units within the Neuss region of North-Rhine Westphalia, Germany. Moderate bivariate relationships were found between all IQ subtests and the concentration performance variables (d2). A substantial overlap in variance was found between “nonverbal” intelligence and the concentration variables (using multiple regression and discrimination analysis). Error rate on the concentration task was significantly negatively correlated with the IQ variables, the magnitude of the correlation coefficient increasing as a function of the time on the task. Future studies would benefit from comparisons in factor structure similarity between abnormal and normal groups as well as between-clinical groups. At a practical level, the relatively easy use (less complex administration) and less obtrusive (hence, low level of personal threat) and inexpensive procedures of the letter cancellation task makes it a useful, albeit “approximate”, measure of cognitive functioning.

## INTRODUCTION

Several problems are inherent in the psychological assessment of cognitive functioning among clinical groups that include psychiatric and geriatric patients: Most inventories of cognitive functioning are time-consuming, "ego-threatening", emotionally exhaustive, and costly procedures. Brickenkamp[1] constructed a letter cancellation task (d2) for assessing short-term concentration that has been widely implemented in psychodiagnostic assessment, essentially as a means of selection in educational and occupational settings and more recently in clinical situations. The task resembles a choice-reaction time paradigm and, despite some limitations, offers an objective measure of speed, accuracy, and fluctuation in cognitive performance.

Negligible correlations have been reported between the Wechsler Adult Intelligence Subtests (WIP) and working pace (GZ) on the d2 concentration task[1]. Results from clinical studies, however, involving diverse psychiatric groups, have demonstrated significant positive correlations between concentrative ability and the four WIP subscales, especially the nonverbal intelligence subtests: picture completion and block design[2,3]. Similarly, Kühnlein et al.[4] obtained moderate positive correlation coefficients between performance on the d2 and Raven's Standard Progressive Matrices for an abnormal group (neurotics and patients with diffuse psycho-organic syndrome). They concluded that persons showing psychological disorders were inclined to show a significant contribution from intelligence on tasks involving concentration.

In an attempt to validate a test battery (comprising an intelligence test WIP, Benton's Visual Retention Test, a verbal-visual retention task, and Brickenkamp's d2 "Aufmerksamkeit-Belastungstest") among a heterogeneous clinical group, Hahlweg[5] applied principal component factorial analysis and extracted two major factors: "general intellectual ability" (high positive loadings on all four IQ subtests, working pace and correct responses yielded on the d2 concentration and the Benton task), and a second factor (positive loadings on block design and accurate responses on the Benton Test, as well as negative loadings on percentage error and fluctuation in response rate on the d2). A further study involving 90 psychiatric patients[2] used the German Adult Wechsler Intelligence Test (HAWIE) and the d2 concentration task. Factor analysis of the task variables revealed that for neurotic patients, performance variables on the concentration task constituted a distinct factor, while for individuals suffering from psycho-organic disorders, a relatively pure factor was extracted that loaded on the intelligence subtests, symbol substitution and arithmetic, and the d2 variable, working pace (total number of correct and error responses), which was essentially associated with the preferred rate of working. Error rate and fluctuation in response rate (both d2 variables) and digit retention, formed an independent factor "concentrative ability".

Where differences have been found in the association between intelligence and performance on a letter cancellation task, a theoretical explanation would be valuable. Brebner[6] introduced the concepts of stimulus analysis and response execution to distinguish between the two motor performance responses. It could also be argued that facets of intelligence that focus on movement responses (nonverbal intelligence) are more likely to be strongly associated with performance on tasks involving speed on psychomotor components, such as the letter cancellation task. Verbal intelligence tasks presumably depend more on cognitive processing, involving stimulus analysis and less with response execution, and while being related to concentration tasks, the relationship will not be as pronounced as in nonverbal tasks.

This study examines the interrelationship between intelligence and concentrative ability for a group of psychiatric patients. Several hypothesis can be tested: (1) performance on the letter discrimination and cancellation task (d2) will be influenced by intelligence niveau, (2) the magnitude of the relationship between IQ and concentrative ability will be higher for operational (nonverbal) intellectual ability, and (3) by examining the error rate in discrete phases of the cancellation task, the IQ-concentration link will be moderated by effects of fatigue.

## METHOD

A group of 85, predominantly psychotic, male patients with a mean age of 32 years, either in the Sheltered Workshop (GWN) or neurological-psychiatric institutionalized care units within the Neuss region of North-Rhine Westphalia, were required to complete an array of psychological tests intended to promote occupational rehabilitation.

A letter cancellation task[1] was administered to patients who were required to delete those "d"s that were accompanied by a total of two strokes, ignoring all other letters (e.g., "b"s and "p"s) or combinations of strokes ("d"s with one or more than two strokes) in any particular line series. At regular 20-sec intervals, respondents were required to stop and move on to the next line, thus allowing for accurate monitoring of working pace (GZ, 357.64 letters SD 116.96; s.e. 13.51, range 77–607), accuracy of responding (GZ-F 324.03 correct responses SD l07.07, s.e. 12.36, range 73–554), error rate (F, 33.95 SD 36.96., s.e. 4.27, range 1–215), percentage error (9.84 SD 8.73, s.e. 1.01, range 0–38), and fluctuation (14.37 SD 5.87, s.e. 0.68, range 6–34) in response rate. Overall, the task required 280 sec for completion.

Finally, a reduced version of the WIP was used[3], including two verbal (information and similarities) and nonverbal intelligence subtests (picture completion and block design). The sample mean raw scores for the subscales were not very different from the population norms (information, 11.81 S.D 6.01; similarities, 13.32 SD 5.99; picture completion, 10.35 SD 2.93; block design, 19.44 SD 9.12, n = 75).

## RESULTS

Pearson product-moment correlations were obtained between the four Wechsler subscales and the five d2 concentration variables (Table 1). The correlation coefficients were all statistically significant indicating considerable overlap between intellectual functioning and concentrative ability. The average correlation was 0.36 for both verbal IQ subtests (information and similarities).

**TABLE 1**
**Correlation Coefficients (Pearson's r) Between the Four Wechsler Subscales (Information, Similarities, Picture Completion, and Block Design) and the Five Attentional (d2) Variables**

| | Information | Similarities | Picture Completion | Block Design |
|---|---|---|---|---|
| GZ | 0.29* | 0.26* | 0.32** | 0.48*** |
| GZ-F | 0.42*** | 0.39*** | 0.47*** | 0.62*** |
| F | –0.29* | –0.33** | –0.34** | –0.28* |
| F% | –0.44*** | –0.45*** | –0.46*** | –0.49*** |
| SB | –0.36** | –0.38*** | –0.35** | –0.25* |

*$p < 0.05$, **$p < 0.01$, and ***$p < 0.001$.

The magnitude of the mean correlation was somewhat higher for the nonverbal intelligence subtests, picture completion (average | r | = 0.39) and block design (mean r –0.42)

A multiple regression analysis, using the frequency of correct responses on the d2 task as the dependent variable, and the four IQ subtests as predictor variables, revealed that the intelligence scores accounted for 25% of the concentrative task variance (R = 0.50, F = 7.74). On the other hand, the contribution from the verbal IQ scales information (t = 0.85) and similarities (t = 0.99) were not statistically significant, while both nonverbal IQ subtests, picture completion (t = 3.74, $p < 0.001$) and block design (t = 2.60, $p < 0.01$), were.

In addition, multiple regression discrimination analysis comparisons were made to examine the simultaneous effect of all IQ subtests as predictors of accuracy of responding. Three groups were generated on the basis of high, intermediate, and low scoring on the d2 variable (d2+ SW > 115, d2– SW < 71, and intermediates, SW 93–96). The first discriminant function emerged as statistically significant (L = 0.31, Rao's R = 2.56, df 8.26, $p < 0.03$), thus rejecting the multivariate generalized hypothesis of no differences in IQ profiles between groups. Univariate F-tests demonstrated that these differences were limited to the nonverbal IQ subscales, picture completion (F = 3.47, $p < 0.05$) and block design (F = 6.67, $p < 0.01$), consistent with the regression analyses.

The distribution of errors (omissive and commissive) across the d2 task was examined in relationship to the intelligence subtest scores. Table 2 reveals the bivariate correlations between IQ and errors yielded in the early (9.1 SD 9.4), middle (14.0 SD 15.8), and final (10.4 SD 12.5) stages of the concentration task.

**TABLE 2**
**Interrelationship Between Wechsler Intelligence Subtests and the Distribution of Errors (Commissive and Omissive) over Three Periods, Ft1, Ft2, and Ft3 (Beginning, Middle, and End Phase, Respectively)**

| | Information | Similarities | Picture Completion | Block Design | Xr |
|---|---|---|---|---|---|
| Ft1 | –0.22 | –0.21 | –0.35*** | –0.24* | –0.26* |
| Ft2 | –0.28* | –0.32** | –0.33** | –0.26* | –0.30** |
| Ft3 | –0.32** | –0.37*** | –0.32** | –0.31** | –0.33** |

Note: Xr represents the mean correlation coefficient across all subtests.

**p* < 0.05, ***p* < 0.01, and ****p* < 0.001.

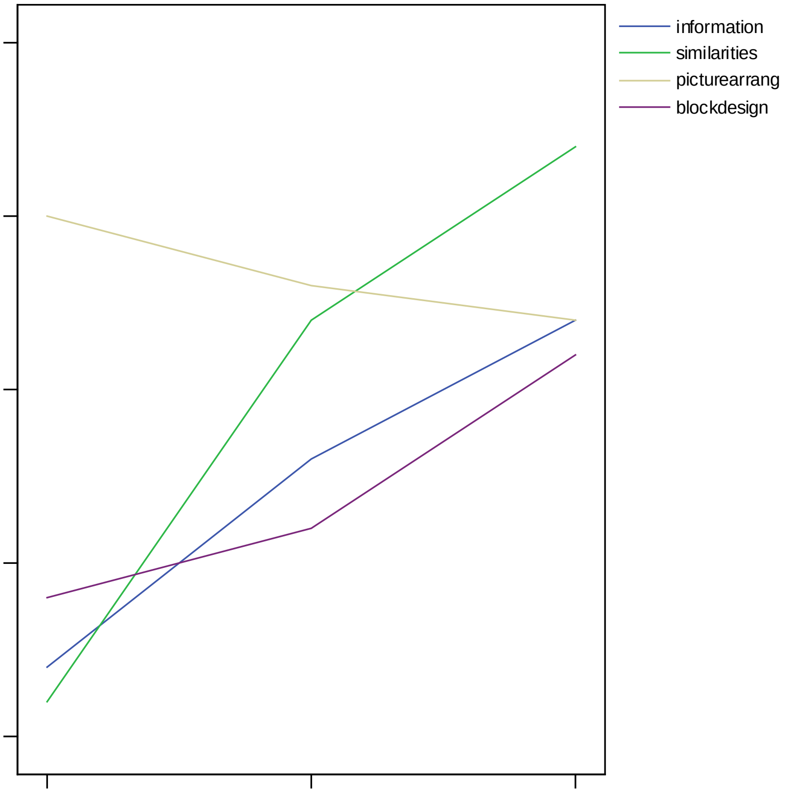

**FIGURE 1.** The magnitude of the correlation coefficients from early, intermediate, and late trials of the letter cancellation task.

## DISCUSSION

The relationship between intelligence and concentration (H1) was quite substantial in contrast to those reported by Brickenkamp[1]. They are, however, of similar order of magnitude to the correlation coefficients obtained using diverse clinical groups[2,4,5] (Fig. 1). The highest correlations were found between d2 performance and block design (form perception or combinative ability) and picture completion

(ability to distinguish essential from unessential details), both subtests of a nonverbal kind. The multivariate statistical analyses substantiated the importance of the operational intelligence subtests, picture completion, and block design as good predictors of concentrative ability (H2). Overall, these findings suggest that intellectual functioning, while unrelated to concentration among normal populations, would appear to be closely interrelated for psychologically "impaired" individuals, and this relationship particularly pronounced for nonverbal intelligence.

By dividing the d2 task into three equivalent periods, it was possible to analyze differential trends in the relationship between IQ and distribution of errors: All WIP subscales, with the exception of picture completion, showed increasingly higher correlations between accuracy of responses and intelligence as the task progressed. It may be that this corresponds to the effects of fatigue, which accumulate as the task prolongs (H3). More specifically, individuals with lower intellectual functioning are inclined to display higher rates of errors resulting from reduced concentration across time.

Performance of the d2 (visual concentrative task) appears to possess a marked motoric component similar to the cognitive tasks entailed in nonverbal intelligence subtests (based on speed and accuracy of response). This was supported by the inferior predictive power of the verbal intelligence subscales scores.

Several reasons can be offered for the strong association between intellectual functioning and concentration among psychiatric groups. It may be that previous studies have tended to focus on university students — all with well-above-average intelligence — and this narrower range of IQ and concentrative ability may have attenuated the magnitude of the correlation coefficient. An alternative is that poor performance on both tasks may reflect a general cognitive impairment of inefficiency resulting from the illness itself, so that the common variance between cognitive tasks reflects the secondary or nonspecific effects of the disorder itself.

Future studies would benefit from comparisons in factor structure similarity between abnormal and normal groups as well as between-clinical groups. At a practical level, the relatively easy use (less complex administration) and less obtrusive (hence low level of personal threat) and inexpensive procedures of the letter cancellation task makes it a useful, albeit "approximate", measure of cognitive functioning. Certainly, in situations in which linguistic ability is limited (preschool children, geriatric groups, and migrants with scarce knowledge of the host nation's language), such a speedy measure of intellectual functioning would seem valuable. In other clinical settings, Almeida and Tamai[7] used, among other measures, a letter cancellation task as a quick and effective way of evaluating the effectiveness of medical treatment among congestive heart failure patients on cognitive functioning.
